# Advancements in Acute Pulmonary Embolism Diagnosis and Treatment: A Narrative Review of Emerging Imaging Techniques and Intravascular Interventions

**DOI:** 10.3390/jcdd12090333

**Published:** 2025-08-29

**Authors:** Michaela Cellina, Matilde Pavan, Niccolò Finardi, Francesco Cicchetti, Maurizio Cè, Pierpaolo Biondetti, Carolina Lanza, Serena Carriero, Gianpaolo Carrafiello

**Affiliations:** 1 Radiology Department, ASST Fatebenefratelli Sacco, Ospedale Fatebenefratelli, Piazza Principessa Clotilde 3, 20121 Milan, Italy; 2 Postgraduate School in Radiodiagnostics, Università degli Studi di Milano, Via Festa del Perdono 7, 20122 Milan, Italy; matilde.pavan@unimi.it (M.P.); niccolo.finardi@unimi.it (N.F.); francesco.cicchetti@unimi.it (F.C.);; 3 Radiology Department, Fondazione IRCCS Ca’ Granda Ospedale Maggiore Policlinico, Via Francesco Sforza 35, 20122 Milano, Italy; maurizioce.md1@gmail.com (M.C.); carolina.lanza@policlinico.mi.it (C.L.); serena.carriero@policlinico.mi.it (S.C.)

**Keywords:** pulmonary embolism, photon counting CT, DECT, mechanical thrombectomy, catheter-directed thrombolysis

## Abstract

Acute pulmonary embolism (APE) represents a significant cause of morbidity and mortality worldwide, requiring rapid and precise diagnosis and effective therapy strategies. Computed Tomography Pulmonary Angiography (CTPA) is currently the gold standard technique for diagnosing PE; however, it presents some disadvantages, including limited sensitivity in detecting sub-segmental emboli and contrast-related risks. Recent advancements in imaging technologies, including Dual-Energy Computed Tomography (DECT) and Photon Counting (PC), offer improved sensitivity and specificity for APE and perfusion abnormalities detection. Digital Dynamic Radiography (DDR) perfusion imaging represents a novel imaging that allows pulmonary perfusion assessment without contrast medium administration, able to detect anomalies at the patient’s bedside, representing a promising advancement, particularly for critically ill or contrast-allergic patients. In parallel, interventional radiology has become integral to APE management, particularly for high-risk and intermediate–high-risk patients, with evolving intravascular treatment techniques such as catheter-directed thrombolysis, mechanical thrombectomy, and thrombus aspiration. This narrative review provides an overview of the latest developments in APE diagnostic imaging and interventional radiology, contextualizing them within current guideline recommendations for endovascular treatment.

## 1. Introduction

Acute pulmonary embolism (APE) is a serious condition characterized by the obstruction of blood circulation in the pulmonary arteries or their branches, mainly caused by a blood clot, which typically originates in the deep venous system of the legs [1]. This condition places a significant strain on healthcare systems, with an estimated occurrence of 39 to 115 cases per 100,000 individuals annually in the United States, and a steady rise over the last ten years [2]. Additionally, the literature reports that around one-third of patients with APE will experience a recurrence within a decade [2].

Although APE is the third leading cause of cardiovascular-related deaths, after coronary artery disease and stroke [3], estimating the precise case/fatality rates for PE can be challenging, with a mortality rate that varies between 60% to less than 1% [4]. This variability highlights the need for effective and timely diagnostic and therapeutic strategies, with early detection being crucial. Early and accurate diagnosis is essential for prompt, life-saving therapy while avoiding unnecessary anticoagulation treatments in patients without APE. The clinical presentation of APE is highly variable and often nonspecific, overlapping with other cardiopulmonary conditions such as pneumonia, acute coronary syndrome, and heart failure [5], making diagnosis highly dependent on imaging techniques [6]. However, despite the often non-specific clinical signs, the introduction of clinical prediction tools, such as the Wells Score, the Geneva Score and the YEARS algorithm, has significantly aided clinicians in identifying patients who are more likely to have APE and should therefore undergo further diagnostic imaging [7].

Incidental APE detection on CT scans across different populations is approximately 2.6%, with elevated rates in cancer patients (up to 5%) and hospitalized individuals [5], with most incidental APEs clinically relevant [5].

Throughout the years, computed tomography pulmonary angiography (CTPA) has emerged as the reference standard imaging method for diagnosing APE [2]. CTPA is a non-invasive procedure characterized by high sensitivity (96–100%) and specificity (89–98%) [8], but despite its accuracy, CTPA has drawbacks, as it heavily depends on optimal contrast opacification of the pulmonary arterial tree, which can be affected in patients with poor cardiac output, improper timing of contrast bolus and other technical issues, leading to non-diagnostic results. Additionally, the use of ionizing radiation and iodinated contrast media presents cumulative risks, especially in younger patients, pregnant women, and those with existing renal issues or contrast allergies [9]. These constraints underscore the necessity for ongoing enhancement of diagnostic approaches and the investigation of new imaging techniques.

Recent imaging methods have proven valuable for diagnosing APE. Dual-Energy CT (DECT) enables the generation of pulmonary perfusion maps, offering functional data alongside anatomical details, enhancing diagnostic precision, while minimizing the volume of contrast agent [10,11]. Likewise, photon-counting detector CT (PC) demonstrates distinct advantages, such as decreased radiation exposure and minimized contrast agent needs, while maintaining superior image quality [12]. Additionally, Dynamic Digital Radiography (DDR) is emerging as a valuable non-invasive method for evaluating pulmonary function and blood flow, demonstrating a strong ability to identify chronic thromboembolic pulmonary hypertension, akin to ventilation/perfusion scans [13].

Along with these imaging innovations, the management of APE is also evolving, with the emergence of risk-stratification tools and interventional radiology-based treatments [14]. These techniques, including catheter-directed thrombolysis and mechanical thrombectomy, have emerged as effective alternatives, especially for patients with contraindications to systemic thrombolysis or those who have not responded to it [15]. Recent studies have not only demonstrated the safety and efficacy of these interventional approaches but also aim to further evaluate the role of interventional radiology for intermediate–high-risk APE patients, expanding the therapeutic indications of these techniques [16].

This review explores emerging imaging and interventional techniques that are playing an increasingly important role in both the diagnosis and treatment of APE, with a focus on their recent developments and potential clinical applications.

## 2. Search Strategy and Methodology

We conducted a review of the literature by searching the PubMed database up to May 2025 using the following key terms, individually and in combination: ‘pulmonary embolism’, ‘computed tomography pulmonary angiography’, ‘dual-energy CT’, ‘photon-counting CT’, ‘dynamic digital radiography’, ‘catheter-directed thrombolysis’, ‘mechanical thrombectomy’, ‘low-dose systemic thrombolysis’ and ‘artificial intelligence’. Additional relevant articles were identified through manual reference checks of selected papers and recent guidelines.

## 3. Diagnostic Imaging

Recent advances in imaging technology have emerged to address some of the inherent limitations of CTPA, which, despite these developments, remains the current gold standard for the diagnosis of APE.

### 3.1. High-Pitch CT

High-pitch helical CT scanning enables rapid acquisition of thoracic images, which is particularly useful in the emergency setting for patients who are dyspneic or unable to cooperate. A recent study analyzed 81 normal-weight patients referred for CTPA for suspected APE: forty-one patients underwent the high-pitch CTPA protocol, while 40 underwent the standard CTPA protocol, both on a second-generation dual-source CT scanner [17]. The key differences between the protocols included the scan pitch (3.2 for high-pitch vs. 1.2 as a standard), scan time (<1 s for high-pitch vs. ~2 s as standard), breathing instructions (free-breathing for high-pitch vs. breath-hold as a standard), and contrast medium volume (20 mL for high-pitch vs. 50 mL). The detection rate of APE was also similar in both groups, and subjective image quality was rated as good to excellent (rating score 1) in over 90% of all exams for both protocols. Contrast opacification was systematically above 250 HU in the high-pitch group, which is considered sufficient for differentiating clots from surrounding tissues. High-pitch CTPA with ultra-low contrast volume resulted in significantly lower mean contrast opacification and noise values in all segmental pulmonary arteries compared to standard CTPA. The median effective dose was significantly lower for high-pitch CTPA (1.04 mSv) than for standard CTPA (1.49 mSv) (*p* < 0.0001), with a dose saving of approximately 30% [17]. The high-pitch mode’s short acquisition time can also mitigate or avoid motion artifacts.

### 3.2. Dual Energy CT

#### 3.2.1. Principles of DECT

DECT relies on the differences in the photoelectric effect and Compton scattering at different energetic levels to generate material-specific maps for the characterization of different tissues and components. Several dual-energy acquisition techniques are currently in clinical use, including dual-source systems, rapid kVp-switching single-source scanners, and layered detector configurations.

Post-processing techniques enable the generation of virtual monoenergetic images (VMIs), virtual non-contrast images (VNC), and iodine distribution maps, all of which contribute to enhanced diagnostic confidence and potentially reduce the need for additional imaging [18]. Iodine maps derived from DECT datasets allow for the visualization of regional pulmonary perfusion by quantifying iodine distribution within the lung parenchyma [19]. these color-coded blood volume maps can reveal perfusion defects corresponding to embolized pulmonary arterial territories, supporting or clarifying vascular findings [20]. DECT integrates both anatomic and functional assessments: the CT angiogram visualizes the clot anatomically, while the iodine map reveals the resulting perfusion deficit, similar to what is seen in a nuclear perfusion scan. This allows for an improved reader detection of segmental and sub-segmental emboli and enables functional assessment of perfusion [21].

#### 3.2.2. Accuracy of DECT

Thieme et al. [22] examined the diagnostic accuracy of DECT perfusion imaging in relation to perfusion scintigraphy. They found a sensitivity and specificity of 75% and 80% per patient, respectively, and a good agreement with scintigraphic findings. In a later investigation [23], the authors explored the diagnostic performance DECT iodine maps (which analyze perfusion defects) against SPECT/CT, finding a sensitivity of 76.7% and a specificity of 98.2%. When evaluating a consensus interpretation of all imaging techniques as the reference standard, DECT-CTPA exhibited 100% sensitivity and specificity in identifying APE, surpassing the combination of SPECT/CT and ventilation scintigraphy (85.7% sensitivity, 87.5% specificity). Grob et al. [24] conducted a prospective study comparing subtraction CT and DECT iodine maps to conventional CTPA. DECT achieved a specificity of 95% and a sensitivity of 85%, which was not significantly different from that of CTPA alone (80% sensitivity).

A meta-analysis by Monti et al. [25] assessed the diagnostic performance of DECT for APE detection, focusing exclusively on lobar and segmental emboli. The analysis stratified results based on different DECT post-processing techniques: linear blending (LB) alone showed a pooled sensitivity of 0.87 (95% CI: 0.76–0.95) and specificity of 0.93 (95% CI: 0.87–0.97) across six study components involving 348 patients; LB combined with iodine maps showed a slightly improved sensitivity of 0.89 (95% CI: 0.82–0.94) and specificity of 0.90 (95% CI: 0.86–0.94), based on 14 study components and 1007 patients; the combination of LB, iodine maps, and VMIs achieved a sensitivity of 0.90 (95% CI: 0.70–0.98) and specificity of 0.90 (95% CI: 0.77–0.97), although data were limited to two studies totaling 144 patients. Despite these encouraging results, the meta-analysis concluded that DECT, regardless of the reconstruction approach used, did not demonstrate a substantial diagnostic advantage over conventional single-energy CT (SECT) [26].

Finally, Foti et al. [27] prospectively evaluated the diagnostic performance of venous-phase DECT in oncology patients, using standard CTPA as the reference standard. Among 61 patients (30 with confirmed APE), venous phase DECT demonstrated a per-patient sensitivity of 90.0% and specificity of 100% for both readers; lung perfusion imaging showed lower performance, with a per-patient sensitivity of 73.3% and specificity of 67.7%.

In addition to detection, DECT offers further characterization and prognostic information; perfusion defect scoring has been suggested to measure APE severity, and research has indicated a link to right ventricular dysfunction and negative outcomes [28,29]. It provides considerable benefits concerning the creation of VNC and virtual VMIs from one contrast-enhanced acquisition. VNC images are produced by subtracting the iodine-attenuation component from the contrast-enhanced dataset using material decomposition algorithms, thereby simulating a non-contrast examination [30], and eliminating the need for an additional scan, significantly reducing radiation exposure, particularly important for young patients and those undergoing repeated imaging. Additionally, VNC images enhance the assessment of incidental pulmonary nodules, vascular calcifications, or hemorrhage by allowing a thorough examination of lung parenchyma, calcifications, and mediastinal structures revealed by intravenous contrast.

**Figure 1:**
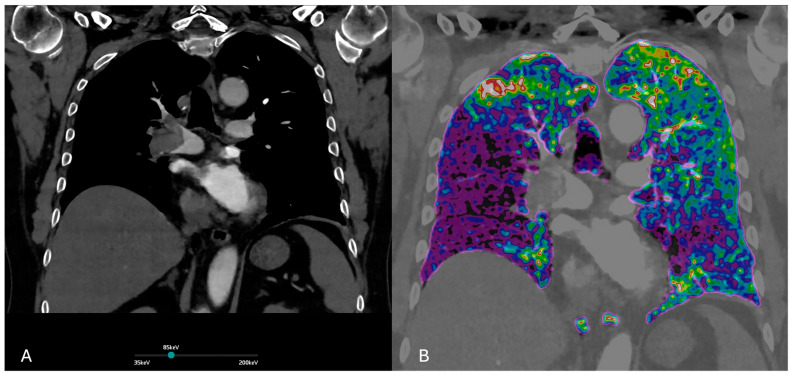
(**A**) Dual-energy CT. Virtual monochromatic at 85 keV image enhances vascular contrast while minimizing noise and beam-hardening artifacts. This reconstruction optimizes visualization of pulmonary embolism within the pulmonary arteries. (**B**) Iodine perfusion map demonstrates the regional lung perfusion. Areas of reduced iodine uptake (shown in purple) correspond to perfusion defects due to embolic obstruction of the pulmonary arteries.

**Figure 2:**
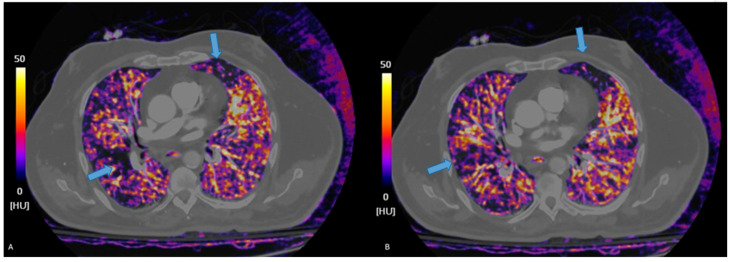
Iodine maps help detect segmental perfusion deficits, hard to detect on traditional CTPA, visible as defects in iodine contents (blue arrows, (**A**,**B**)), whiting the lung parenchyma.

VMIs are images synthesized at different energy levels, usually ranging from 40 to 190 keV (Figure 1 and Figure 2). VMIs at low-keV levels (e.g., 40–60 keV) increase the attenuation of iodine within the pulmonary arteries, improving the contrast-to-noise ratio and the conspicuity of emboli in pulmonary arteries, also compensating for suboptimal contrast opacification due to low cardiac output, poor injection technique, or delayed bolus timing. VMIs also allow for the reduction in the administered contrast medium amount without compromising vascular visualization, particularly useful in patients with impaired renal function.

Finally, the use of high-energy VMIs (e.g., 100–140 keV) can mitigate beam-hardening artifacts from dense contrast or metallic implants, improving overall image quality.

### 3.3. Photon-Counting CT

#### 3.3.1. Principles of Photon Counting

PC is a new imaging technique that greatly improves traditional CT and depends on energy-integrating detectors (EIDs). PC provides enhanced spatial and contrast resolution, lower electronic noise, and the possibility for a decrease in radiation dose [19,31]. The key distinction lies in the use of PC Detectors (PCDs), which work in a completely different way compared to EIDs: while EIDs convert X-rays into light, and then converts this light into electrical signals using a photodiode [32], PCDs, made of special materials like cadmium telluride or silicon, directly convert X-rays into electrons and receive them as electrical signals. PCDs count each photon and measure its energy by sorting it into multiple energy bins (typically three to eight) based on the height of the generated electrical pulse, thereby filtering out electronic noise below a certain threshold. This energy-resolving capability enables accurate multi-energy imaging, overcoming the dual-energy limitations of current spectral CT systems [10]. It allows the decomposition of materials using the energy-dependent attenuation characteristics of each voxel, producing basis image maps that measure substances such as iodine or calcium. These maps are applicable for generating VMIs, VNC images, or images specific to materials [31]. PC additionally facilitates quantitative imaging by delivering physical characteristics at the voxel level, enabling more precise measurement of contrast agents compared to DECT.

PCDs enable a significant reduction in contrast medium administration and radiation dose for diagnosing APE compared to conventional EID-CT while maintaining good to excellent image quality [32].

#### 3.3.2. Diagnostic Accuracy of of Photon Counting-CTPA

Pennenbecker et al. [12] found that PC-CTPA offered better subjective and objective image quality compared to SECT, even with an ultra-low contrast and radiation dose protocol, enabling a 50% reduction in contrast medium and a markedly lower effective dose (1.4 mSv versus 3.3 mSv). In a different study, the authors [33] showed that PC scans provided better subjective and objective image quality than EID images and a 28% decrease in radiation dose when compared to corresponding EID-CTPA, with enhanced CT attenuation in all examined pulmonary vessels.

**Figure 3:**
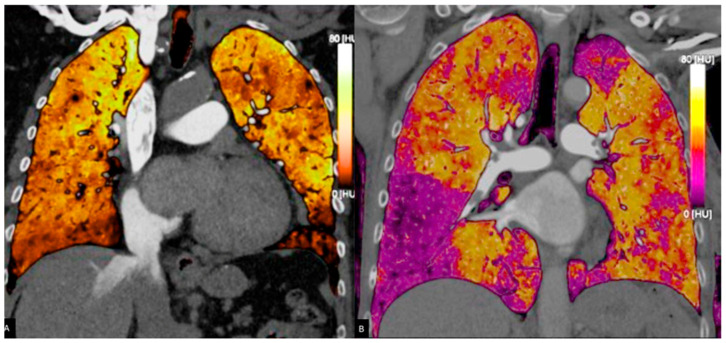
Pulmonary blood volume map derived from PC acquisition, showing iodine distribution. The perfusion is almost homogeneous in (**A**), whereas in (**B**), perfusion deficits are bilaterally present. Areas of reduced perfusion appear in darker shades (**B**), assisting in the identification of embolic territories and functional assessment of the pulmonary circulation.

Morevoer, when working with PC, VMIs help APE diagnosis by optimizing contrast resolution and reducing artifacts [34]. Lower keV VMI reconstructions (40–50 keV) improve vascular attenuation in pulmonary arteries, increasing iodine signal intensity and enhancing thrombus detection sensitivity [35]. This is particularly useful for subsegmental APE identification, where traditional CT may struggle due to limited contrast resolution (Figure 3). VMI generation allows a significant reduction in radiation dose by 46–47% compared to SECT.

Although 40 keV VMIs achieve peak signal-to-noise ratios, 50 keV balances noise reduction and artifact mitigation, with studies demonstrating superior APE visibility at this energy level due to fewer beam-hardening artifacts [35]. Additionally, VMIs enable diagnostic salvage of suboptimal enhanced studies through contrast boost effects, maintaining diagnostic confidence even in technically challenging cases [35].

PC CTPA is now being used routinely in clinical settings, with continuing research aiming to evaluate its diagnostic performance compared with traditional CTPA. Recent prospective investigations have shown that PC produces 95–100% of tests classified as diagnostic for PE, with some series reporting no non-diagnostic scans. In most situations, subjective image quality was rated excellent or good, exceeding EID-CT and DECT [12,33]. Furthermore, PC improves contrast-to-noise and signal-to-noise ratios, reduces motion aberrations, and increases CT attenuation in pulmonary arteries, all of which are crucial for effective PE identification. However, most published studies use diagnostic yield and picture quality as surrogates for accuracy, with few reporting sensitivity and specificity against a reference standard [12,33].

In a recent prospective study, Remy-Jardin et al. [36] found a high diagnosis rate (95.3%) for PC scans, since most non-diagnostic instances, mostly due to inadequate vascular opacification, could be efficiently rectified using low-energy VMI reconstructions. Fletcher et al. [37] found that PC’s greater spatial resolution and contrast-to-noise ratio could provide similar or even better diagnostic accuracy for APE than DECT.

### 3.4. Dynamic Digital Radiography

#### 3.4.1. Principles of Dynamic Digital Radiography

DDR is an emerging low-radiation method for assessing pulmonary perfusion that applies a flat-panel detector and a conventional radiography system with a pulsed X-ray generator, allowing for the capture of sequential high-resolution images over time., creating a dynamic sequence that represents motion and functional changes, such as airflow and blood flow in the lungs.

**Figure 4:**
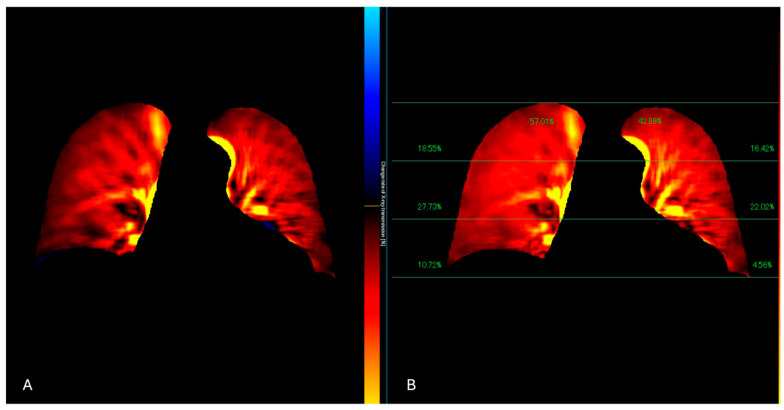
The perfusion algorithm uses the reference frame subtraction method to analyze the degree of pixel value change from the baseline timing (end-diastolic phase) (**A**). After removing the respiratory-related temporal changes in pixel values using a band-pass filter, a color-coded map representing the pixel value change rate and a map showing the contribution of the different lung areas to the global perfusion are obtained (**B**).

**Figure 5:**
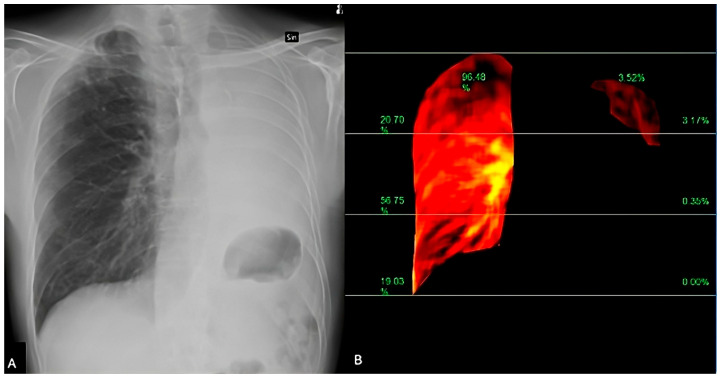
DDR perfusion assessment of a lung cancer patient, presenting dyspnea and raised D-dimer values. The traditional X-Ray (**A**) shows a pleural effusion extending to the apex. The perfusion study (**B**) demonstrated a regular perfusion of the right lung parenchyma and left apex.

Patients are imaged in a standing or supine position over a breath-hold of 6 s, and the obtained images are processed to highlight temporal changes in pixel intensity corresponding to pulmonary blood flow from the baseline timing (end-diastolic phase) [38] (Figure 4 and Figure 5). The resulting images reveal blood flow dynamics within the lungs and a color-coded map that represents the lung perfusion [39].

APE is visible on DDR examinations as well-defined triangular or wedge-shaped perfusion defects, similar to those observed in traditional imaging modalities such as lung perfusion scintigraphy [40].

DDR obtains lung perfusion without utilizing intravenous contrast media, providing a secure option for patients with contraindications to contrast agents and presenting benefits regarding radiation exposure and patient comfort. The low radiation doses (0.2 mSv) compared to traditional imaging techniques make DDR a promising option for functional analysis of pulmonary circulation, especially in patients who require repeated imaging or have limited tolerance for more invasive procedures.

The availability of DDR portable equipment allows the acquisition also at the patients’ bed, speeding up the workflow in the emergency setting.

#### 3.4.2. Accuracy of Dynamic Digital Radiography

Studies have demonstrated the efficacy of DDR in detecting chronic thromboembolic pulmonary hypertension (CTEPH), a pathological condition related to unresolved pulmonary embolism. Research indicates that DDR has comparable sensitivity and specificity to ventilation/perfusion (V/Q) scanning in identifying CTEPH, suggesting its potential as a reliable diagnostic tool in clinical practice [13].

The main advantages of the different imaging techniques are listed in Table 1.

## 4. Artificial Intelligence

### 4.1. Automated Detection

Artificial intelligence (AI) is transforming medical imaging and diagnostics, significantly enhancing precision and efficiency. Recent advancements have focused on developing AI algorithms that efficiently and accurately evaluate medical images [41]. Specifically for APE, AI improves the interpretation of imaging studies such as CTPA, aiding radiologists in detecting subtle PE signs that could otherwise be missed or misinterpreted [42]. By analyzing vast amounts of imaging data, these systems significantly advance CTPA-based APE detection, leading to better diagnostic performance [43]. While diagnosing APE remains challenging due to other conditions that can mimic its appearance and variability in imaging interpretation, AI, particularly deep learning (DL), has emerged as a promising addition to traditional diagnostic pathways [44].

Previous studies utilized machine learning (ML) to create Computer-Assisted Detection (CAD) programs that supported radiologists in identifying APE. Although CAD software exhibited low sensitivity and specificity and necessitated manual verification by radiologists, its incorporation into radiologist workflows greatly enhanced detection rates [45]. Recent deep learning algorithms, particularly Convolutional Neural Networks (CNNs), have demonstrated high accuracy in identifying and localizing pulmonary emboli on CTPA images. These networks analyze different image features, such as shapes, edges, and textures, and gradually learn to identify more complex patterns, like clots inside pulmonary arteries [46,47].

Different AI models have been explored for the APE detection on CTPA [41]. Among the most common AI architectures are U-Net variants, which are convolutional neural networks originally developed for biomedical image analysis [44]. In APE detection, U-Net-based models are used to highlight areas likely to contain emboli by learning spatial patterns across CT slices. These networks can be implemented in different formats: 2D models analyze each slice independently; 2.5D models examine a few adjacent slices simultaneously to capture limited depth information; and 3D models process the entire volumetric scan, providing a more comprehensive spatial understanding of the pulmonary vasculature.

To enhance the performance of these base models, researchers often integrate DenseNet or ResNet components [48], which are DL architectures known for their ability to extract many additional features from medical images. These hybrid models frequently use transfer learning approaches, meaning they are partially pre-trained on large general image datasets and then fine-tuned on APE-specific scans. In some cases, attention mechanisms are added to guide the network’s focus toward areas most likely to contain emboli, improving both accuracy and interpretability [49].

Another important class of models focuses on object detection, which aims to locate and mark the position of emboli within the pulmonary arteries. Examples include Faster R-CNN and Mask R-CNN [49], which generate bounding boxes around suspected emboli by scanning the image for candidate regions.

Numerous studies have evaluated the performance of AI algorithms for APE detection using metrics such as sensitivity, specificity, Area Under the Curve (AUC), and accuracy [41,42,46,50,51,52,53,54]. A systematic review and meta-analysis of DL-based AI algorithms for APE detection on CTPA showed a pooled sensitivity of 88% and a specificity of 86% per scan [55]. A recent meta-analysis [56] examined the effectiveness of DL algorithms for identifying APE on CTPA scans. Analyzing data from twenty-four studies encompassing almost 23,000 patients, the research finds that DL models exhibit high diagnostic accuracy. Specifically, U-Net architecture shows higher sensitivity for detecting true positives, while CNN models demonstrate better specificity with fewer false positives.

Specific AI tools have been evaluated and received regulatory approval. In particular, CINA-PE (Avicenna.AI), an FDA-cleared and CE-marked DL-based algorithm, demonstrated high performance in different studies, with a per-case sensitivity and specificity that range between 93.9–91.4% and 94.8–91.5%, respectively, and an AUC-ROC of 0.92 [57,58]. Notably, the algorithm detected 22 out of 29 (76%) APE cases that were not mentioned in the initial clinical report, leading to a reduction in the overall missing rate from 15.6% to 3.8%. Another study showed CINA-PE reduced the incidental APE missing rate from 50% to 7.1% compared to no AI assistance [59].

Another FDA-approved AI model is Aidoc, which has been tested in real hospitals showing high levels of accuracy, with sensitivity reaching 96.8% and specificity up to 99.9% [60].

Zsarnoczay et al. [50] analyzed the performance of another Deep Neural Network (DNN)-based prototype algorithm that showed a sensitivity of 84.6% and a specificity of 95.1% for PE detection, with an overall accuracy of 93.8%. This DNN algorithm demonstrated excellent sensitivity for detecting central (100%) and lobar (96.7%) APE and achieved a sensitivity of 72.9% for peripheral APE.

When using AI, common sources of false positives included lung masses, pneumonia, and contrast flow artifacts, while false negatives often involved chronic and subsegmental APE. Detecting peripheral and small emboli poses a significant challenge, and current AI initiatives aim to enhance precision in these regions, while earlier research typically concentrated only on major arteries [61]. A SPE-YOLO model, designed specifically for recognizing small APE, reached a sensitivity of 90.70% and an accuracy of 86.45% on an external validation dataset [62].

Beyond contrast-enhanced CTPA, the possible role of automated APE detection on unenhanced chest CT has also been explored [63]. A prototype AI algorithm utilizing nnDetection, based on the Retina U-Net architecture, demonstrated the potential to detect APE on non-contrast CT scans. This algorithm showed varying sensitivity based on location: 54.5% for central, 81.9% for segmental, and 80.0% for subsegmental APE. While sensitivity on unenhanced scans may be lower than on CTPA, such AI tools could potentially guide the decision on whether a follow-up contrast-enhanced scan is necessary. However, external validation with larger sample sizes is needed to confirm these findings and assess reliability in real-world settings. These AI tools can support the radiologist’s workflow by automatically flagging suspicious cases, generating annotated images with bounding boxes highlighting potential emboli, and therefore prioritizing review cases [57]. In some models, these bounding boxes are intentionally expanded to include surrounding vascular structures, offering the algorithm additional anatomical context to refine its predictions. This approach can improve detection accuracy by helping the AI differentiate emboli from normal variants or artifacts [41].

A summary of the performance of the main DL algorithms is presented in Table 2.

### 4.2. Segmentation and Quantification

Beyond detection, AI also plays an important role in segmenting anatomical structures and embolic material. CNNs are often used for segmenting lung lobes, the heart, and pulmonary arteries, regions critical for accurately localizing and quantifying APE. Common architectures include 2D, 2.5D, and 3D U-Nets, alongside advanced models like Retina U-Net, nnU-Net, and MP-Net, all engineered to delineate features at a pixel level [44]. Segmentation facilitates not only the identification and volumetric analysis of pulmonary arterial thrombi but also of cardiac structures, particularly the right and left ventricles. This capability assists in determining the RV/LV diameter ratio, a widely used prognostic marker in APE reflecting right heart strain [64]. Several AI models have demonstrated the capacity to automate both clot and cardiac segmentation without requiring manual annotation [51]. Nevertheless, clot segmentation can occasionally be incomplete or inaccurate, especially in subsegmental branches. Moreover, certain training datasets may lack the high-quality, voxel-level annotations essential for intricate calculations such as the Qanadli score, a severity metric. An ongoing focus of current research involves addressing these limitations by incorporating larger and more diverse annotated datasets [43].

### 4.3. Prediction

Segmentation is useful to establish the APE severity, including estimating the Qanadli score [44], an index that quantifies the arterial obstruction based on the number and location of occluded segmental branches, and the right ventricle-to-left ventricle diameter ratio (RV/LV ratio) [65]. These are crucial metrics for assessing APE severity and predicting mortality. An AI-based approach has demonstrated the feasibility of estimating the Qanadli score with a coefficient of determination (R2) of 0.717 and the RV/LV ratio with an R2 of 0.723 on the test set. This suggests the potential of AI in providing reliable and useful predictions for these important clinical metrics and potentially promoting the use of the Qanadli score as an APE marker in clinical practice [44].

AI is also a promising tool for outcome prediction and risk stratification [66]. ML models surpass traditional risk scoring systems in risk stratification performance. The integration of different data inputs, such as laboratory results, medical history, and vital signs, makes AI capable of predicting APE probability.

Finally, Natural Language Processing (NLP), a branch of AI that focuses on processing written or spoken language by extracting relevant information from radiology reports, medical history and admission notes, can be used to identify patients with a potential diagnosis [67].

## 5. Therapeutic Strategies

### 5.1. Risk Stratification and Interventional Procedure for Pulmonary Embolism

Risk stratification is a cornerstone of managing APE as it determines the appropriate diagnostic and therapeutic strategies [68] and identifies patients who may benefit from advanced therapies such as catheter-directed treatment (CDT) or mechanical thrombectomy. Risk assessment stratifies patients into high-, intermediate-, and low-risk categories, based on hemodynamic status, RV function, and biomarker evidence of myocardial injury [69]. Among these, high-risk and selected intermediate-risk patients are the most relevant populations for interventional approaches [68,69].

High-risk APE is characterized by hemodynamic instability, including cardiac arrest, obstructive shock (systolic blood pressure <90 mmHg or vasopressor use with signs of end-organ hypoperfusion), or persistent hypotension unresponsive to volume resuscitation [69,70,71]. This population has the highest early mortality and requires urgent reperfusion [68]. Current guidelines from the ACCP (2021) recommend systemic thrombolysis via a peripheral vein over catheter-directed thrombolysis for high-risk PE [72]. However, while systemic thrombolysis remains the recommended first-line treatment, CDT and surgical embolectomy remain critical valid options for patients with contraindications to thrombolysis, failed thrombolysis, or shock likely to be fatal before systemic therapy can act [72].

The ESC 2019 guidelines similarly recommend systemic thrombolysis as first-line therapy, with catheter-based options reserved for selected high-risk patients with contraindications or failure of thrombolysis [71]. In such cases, interventional radiology plays a pivotal role by providing minimally invasive alternatives that rapidly restore pulmonary perfusion and improve right heart function, often with a lower risk of bleeding [69].

Although hemodynamically stable patients may not fulfill the criteria for massive APE, they can exhibit severity markers such as increased troponin or brain natriuretic peptide levels, along with RV dysfunction detected through echocardiography or CTPA [71]. Within this group, intermediate–high-risk APE, defined by combined RV dysfunction and elevated cardiac biomarkers, represents the subgroup most likely to benefit from advanced therapies [16,73].

Systemic thrombolysis is not routinely recommended; however, interventional radiology-guided CDT or mechanical thrombectomy may be considered for patients whose clinical condition deteriorates despite anticoagulation, or for those with persistent signs of RV overload and hypoxia [72]. In such cases, “rescue reperfusion” has garnered clinical support, especially since CDT techniques provide effective reperfusion while minimizing systemic thrombolytic exposure [71]. Recent guidelines from the PERT Consortium (2023) [71] also highlight the potential role of reduced-dose systemic thrombolysis in intermediate-risk PE, especially for patients at elevated bleeding risk, reinforcing the need for individualized decision-making.

Interventional radiology plays a limited role in low-risk APE, which includes hemodynamically stable patients, lack of RV dysfunction, and a PESI class I–II or a simplified PESI score of 0 [71]. These patients are typically treated successfully with anticoagulation alone, and many may be managed in outpatient settings [69].

### 5.2. EkoSonic Endovascular System (EKOS)

The EkoSonic Endovascular System (EKOS) is a directed thrombolysis platform that combines ultrasound energy with low-dose fibrinolytic infusion to accelerate thrombus dissolution. Its efficacy was evaluated through different trials, such as the ULTIMA trial, a randomized controlled trial enrolling 59 patients with intermediate-risk APE, which showed that ultrasound-assisted CDT using EKOS was significantly more effective than anticoagulation alone in reducing RV/LV ratio at 24 h, with no major bleeding events reported [73]. Another study on EKOS was the SEATTLE II study, a prospective multicenter trial involving 150 patients with massive or submassive APE. This study demonstrated a significant reduction in the RV/LV diameter ratio (mean change from 1.55 to 1.13) and a decrease in mean pulmonary artery pressure (from 51.4 mmHg to 36.9 mmHg) following EKOS treatment [74]. However, the significant bleeding rate was 10%. Importantly, this trial lacked a control group, limiting the strength of conclusions regarding efficacy relative to standard care.

The OPTALYSE-PE trial aimed to determine the optimal duration and dose of thrombolytic delivery using EKOS in intermediate-risk patients [75]. This randomized, multicenter trial enrolled 101 patients into four groups receiving different combinations of recombinant tissue plasminogen activator (rtPA) doses (4–12 mg) and durations (2–6 h). All regimens demonstrated rapid improvement in the RV/LV ratio and thrombus burden, with a significant bleeding rate of 4%, which included two intracranial hemorrhages, with one attributed to rtPA. These findings support the feasibility of shorter, lower-dose thrombolytic protocols that may improve safety while preserving efficacy. The SUNSET sPE trial highlighted a different outcome; another randomized study of 81 patients compared EKOS with standard CDT devices and found no significant difference in thrombus score reduction, suggesting that the benefits of ultrasound facilitation may be less pronounced than initially thought. However, the trial was limited by a small sample size [76].

A highly anticipated trial is the HI-PEITHO study, a large, prospective, multicenter, randomized controlled trial designed to compare EKOS-based CDT plus anticoagulation versus anticoagulation alone in 406 patients with intermediate–high-risk APE. The trial’s primary outcomes include PE-related death, recurrence, and cardiorespiratory decompensation at 7 days. The results will significantly influence future guideline recommendations regarding CDT use in intermediate-risk patients [16].

Recent case reports also highlight the clinical applicability of catheter-directed therapies such as EKOS in complex APE scenarios, where systemic thrombolysis poses significant risks. For example, a 50-year-old female patient with May–Thurner Syndrome and extensive thrombotic burden was successfully treated using bilateral EkoSonic Endovascular System (EKOS) catheters without the use of systemic thrombolysis [77]. The patient presented a bilateral submassive pulmonary embolism and a large right atrial thrombus, conditions that, combined with her comorbidities, would traditionally complicate treatment decisions, even if she was hemodynamically stable. EKOS therapy resulted in complete resolution of the atrial thrombus within 30 h, and the patient was discharged home on anticoagulation by day five. This case illustrates how ultrasound-assisted catheter-directed thrombolysis may offer a safer and effective alternative in patients at higher risk of bleeding or those with anatomically complex thrombi. Such real-world outcomes further support the consideration of CDT in intermediate–high-risk PE patients where systemic fibrinolysis is contraindicated or not recommended.

### 5.3. FlowTriever System (Inari Medical)

The FlowTriever System (Inari Medical, Irvine, CA, USA) is a mechanical thrombectomy device using a large-bore catheter, available in 16F, 20F, and 24F sizes, and a 60 mL syringe with a locking mechanism to create a vacuum, designed to remove thrombus from the pulmonary arteries without thrombolytics. It uses aspiration through the Triever catheter (Inari Medical, Irvine, CA, USA) and mechanical engagement tools with self-expanding nitinol disks, offering immediate hemodynamic benefits compared to the gradual improvements seen with CDT. Although technically more demanding, effective outcomes have been favorable, regardless of operator experience.

Significant evidence from various studies highlights the efficacy and safety of FlowTriever across different patient settings. The FLARE study, a prospective, single-arm trial of 106 intermediate-risk PE patients, demonstrated a significant average reduction (25%) in the RV/LV ratio at 48 h post-procedure, along with a low rate (3.8%) of composite adverse safety outcomes and minimal bleeding complications [74]. The FLASH registry, encompassing a larger, real-world cohort of over 800 patients (primarily intermediate- and high-risk APE), reported immediate improvements including a 23% decrease in mean pulmonary artery pressure, enhanced oxygenation, and heart rate stabilization [78,79]. FLASH further confirmed excellent safety, with a 30-day mortality of only 0.9% and major bleeding rates under 1%, supporting FlowTriever as particularly valuable in patients contraindicated to thrombolysis.

**Figure 6:**
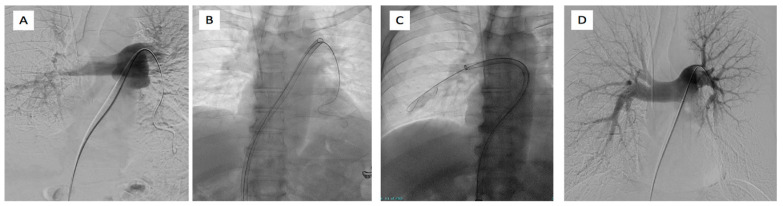
65-year-old male affected by bilateral pulmonary embolism treated with FlowTriever system. (**A**) Left pulmonary artery catheterism and phlebography demonstrated the presence of right main pulmonary artery thrombus and lack of opacification of lower left lobar pulmonary branches. (**B**) Placement of 24F inari device in the left pulmonary artery and (**C**) in the right pulmonary artery with the aspiration of clots. (**D**) Final phlebography demonstrated the patency of the pulmonary artery on both systems with good peripheral vascularization.

In the high-risk APE setting, the FLAME study compared 53 patients treated with FlowTriever (Figure 6) to historical controls, revealing notably lower in-hospital adverse clinical events, primarily driven by an impressively low mortality rate (1.9%) [80].

The PEERLESS study further strengthens FlowTriever’s evidence base as the first randomized controlled trial (RCT) to directly compare two advanced catheter-based therapies, FlowTriever versus CDT, in intermediate-risk APE patients. Enrolling 550 patients, PEERLESS demonstrated a significantly favorable primary endpoint for FlowTriever, primarily driven by fewer cases of clinical deterioration, lower need for bailout therapies, and reduced ICU admissions and lengths of stay [81]. Notably, there was no significant difference between groups in mortality, major bleeding, or intracranial hemorrhage. FlowTriever was also associated with fewer all-cause readmissions within 30 days compared to CDT.

These studies underscore FlowTriever’s capacity for rapid thrombus removal, significant acute improvement in right ventricular function, and an impressive safety profile, making it a compelling therapeutic choice for a broad spectrum of APE patients.

### 5.4. Indigo Aspiration System (Penumbra Inc.)

The Indigo Aspiration System (Penumbra Inc., Alameda, CA, USA) is a mechanical thrombectomy device designed to rapidly remove thrombus in APE using continuous aspiration and mechanical fragmentation. The system features a computer-assisted vacuum thrombectomy (CAVT), regulated by a microprocessor, facilitating precise thrombus extraction while minimizing blood loss. The device comprises aspiration catheters of various sizes (8–12 Fr), a suction pump, and an optional separator wire for enhanced thrombus disruption.

Clinical evaluation through the EXTRACT-PE study—a prospective, multicenter, single-arm trial with 119 intermediate-risk PE patients—demonstrated significant efficacy, showing a mean reduction of 0.43 (27.3%) in the RV/LV ratio at 48 h. Safety outcomes were favorable, with a major adverse event rate of only 1.7%, major bleeding occurring in 1.7%, and no cases of intracranial hemorrhage reported. Notably, 98.3% of patients underwent the procedure without adjunctive thrombolytic therapy. The median procedural duration of 37 min and minimal blood loss further underscored the efficiency and safety of this system [82].

Additional support comes from interim results of the STRIKE-PE trial, a larger prospective, international study evaluating Indigo Aspiration in intermediate- and high-risk APE patients. Interim findings from 150 patients confirmed significant acute hemodynamic improvements, including a 25.7% reduction in the RV/LV ratio and reduced pulmonary artery pressures at 48 h, alongside low complication rates (2.7% composite major adverse events) and notable functional and quality-of-life benefits at 90 days [83]. The ongoing STRIKE-PE trial plans to enroll 600 patients, offering extended follow-up data for up to one year.

Together, these studies position the Indigo Aspiration System as an effective, safe, and versatile mechanical thrombectomy option for managing APE, particularly in populations where thrombolysis is contraindicated or undesirable.

Clinical case reports further emphasize the role of the Indigo Aspiration System in high-risk pulmonary embolism scenarios, particularly where thrombolysis is contraindicated. Angelini et al. [84] described the case of a 75-year-old male presenting with extensive bilateral pulmonary emboli and right ventricular dysfunction, but without hemodynamic instability. Given the patient’s older age and risk of bleeding, catheter-directed aspiration with the Penumbra Indigo system was chosen. A successful removal of 40% of the thrombus burden occurred without any procedural complications, and the patient showed quick clinical improvement, leading to discharge just two days after the intervention. This case exemplifies how aspiration thrombectomy may provide a safe and effective alternative to systemic thrombolysis in intermediate- to high-risk APE patients, especially those with comorbidities that increase bleeding risk. These findings support trial data and highlight the growing role of mechanical thrombectomy in modern APE management algorithms.

### 5.5. Other Devices

Other devices include the AngioJet rheolytic thrombectomy system (Boston Scientific, Marlborough, MA, USA), which utilizes high-pressure saline jets to fragment and aspirate thrombi and allows local thrombolytic injections. The AngioJet system has primarily been assessed in small, retrospective studies involving intermediate- and high-risk APE patients. One retrospective study of 44 patients (21 high-risk and 23 intermediate–high-risk) showed significant short-term clinical and hemodynamic improvements. However, mortality was relatively high (six in-hospital deaths), and complications such as significant bleeding occurred in 4.5% of patients [85]. Another single-center experience with 56 patients noted a significant bleeding rate of 7.1% and mortality of 8.9% [86]. Due to complications like bradyarrhythmia, hemolysis-related acute kidney injury, and hemoptysis, the FDA issued a black box warning for its use in pulmonary arteries, emphasizing the need for careful patient selection and procedure management.

The AngioVac (AngioDynamics, Oakville, ON, USA) is a large-bore (24 Fr) catheter system designed for venous thrombus removal via extracorporeal venovenous bypass. It allows filtration of aspirated thrombus before reinfusion into the systemic circulation. However, the available literature does not provide detailed evidence from specific randomized trials or robust clinical studies conclusively demonstrating its efficacy in treating PE, relying instead on limited single-center experiences and case reports.

The BASHIR endovascular catheter (Thrombolex, New Britain, PA, USA) introduces a unique concept of mechanical expansion and local thrombolysis via an expandable infusion basket with multiple orifices. Preliminary data have shown significant improvements in RV function and thrombus burden with a favorable safety profile [87]. While not yet supported by large-scale randomized trials, its use is increasing in specialized centers.

## 6. Systemic Thrombolysis

The mortality rate from an APE primarily depends on how well the right ventricle (RV) can cope with the sudden strain placed upon it, and quick intervention is vital for APE patients showing signs of RV failure. Anticoagulation treatment is effective in preventing clot progression; however, it does not significantly improve RV function or pulmonary perfusion in the early stages of treatment [88]. Several studies have shown that anticoagulant therapy alone does not lead to marked improvements in RV function or lung perfusion in the initial phase of management [89,90].

Thrombolytic therapy has instead demonstrated more immediate benefits, including significant improvements in both RV function and pulmonary perfusion.

The impact of low-dose systemic tissue plasminogen activator (tPA) thrombolysis on patient outcomes remains a subject of debate, as thrombolytic therapy carries an increased risk of bleeding complications [91].

To minimize this risk while retaining therapeutic benefits, low-dose, prolonged thrombolysis using alteplase or tPA has been proposed as an alternative for patients with intermediate- or high-risk APE [92]. This strategy involves administering reduced doses of tPA over an extended duration, as opposed to conventional full-dose regimens.

Low-dose systemic tPA has emerged as a promising treatment option for APE, particularly in intermediate-risk patients. Compared to anticoagulation alone, this approach seeks to achieve more rapid clot resolution and improved RV function, with a lower bleeding risk than traditional high-dose thrombolytic therapy [93].

Common regimens include a 6 h infusion of low-dose tPA (e.g., 50 mg total, sometimes given as a 10 mg bolus followed by infusion) to balance thrombolytic efficacy with bleeding risk reduction [94]. This administration has proven to be effective in reducing hospital length of stay and decreasing hemodynamic decompensation in intermediate-risk APE patients [95].

## 7. Pulmonary Embolism Response Teams (PERTs)

Many institutions have established Pulmonary Embolism Response Teams (PERTs) to support decision-making in this increasingly complex therapeutic landscape. These multidisciplinary teams typically include pulmonologists, cardiologists, emergency medicine physicians, hematologists, interventional radiologists, cardiothoracic surgeons, and pharmacists. PERTs provide rapid consultation, often via virtual or in-person meetings, and facilitate real-time clinical, imaging, and laboratory data analysis to guide optimal treatment strategies [96].

Their role is particularly vital in intermediate- and high-risk APE cases, or in low-risk patients with complicating factors such as significant comorbidities or contraindications to standard therapies.

Studies assessing the impact of PERT implementation have demonstrated notable benefits. Direct PERT consultation has been associated with reductions in 30-day mortality, hospital length of stay, time to therapeutic anticoagulation, and bleeding complications, even though patients receiving PERT consultation were more likely to undergo advanced interventions such as thrombolysis or thrombectomy [96,97]. The advantages stem from the process of coordinated multidisciplinary decision-making rather than the mere presence of a PERT at the institution. In addition to improving clinical outcomes, PERTs promote the judicious use of resources, such as avoiding unnecessary placement of inferior vena cava (IVC) filters and facilitating patient enrollment into clinical trials [98]. Through registries like KNOCOUT PE [99] and trials such as PE-TRACT [100], PERTs generate high-quality data that may shape future guidelines and standards of care.

## 8. Discussion

The management of APE has evolved remarkably over the past decade, driven by innovations in diagnostic imaging, interventional radiology, AI, and structured clinical management frameworks such as PERTs. While CTPA remains the reference standard for diagnosis [8,9], its limitations, particularly in detecting subsegmental emboli and in patients contraindicated for iodinated contrast, have spurred the development of advanced imaging modalities. Technologies such as DECT, PC, and Dynamic Digital Radiography (DDR) [10,12,13] offer functional insights into pulmonary perfusion and enhanced image quality at lower contrast and radiation doses. However, while promising, most of these technologies require further validation in large, prospective multicenter studies to establish their impact on clinical outcomes, management decisions, and health economics.

Simultaneously, AI has begun to integrate into APE diagnostics, with deep learning algorithms showing high diagnostic accuracy and support in CTPA interpretation. FDA-cleared tools like CINA-PE and Aidoc have demonstrated improved detection rates of APE and potential reductions in missed diagnoses, particularly for incidental and peripheral emboli [55]. AI applications in image segmentation, Qanadli score estimation, and outcome prediction are also expanding, offering future opportunities for comprehensive, automated PE severity assessment.

From a therapeutic perspective, the field of interventional radiology has made substantial progress in addressing high- and intermediate–high-risk APE, especially in patients contraindicated for systemic thrombolysis. Catheter-directed therapies (CDTs) such as the EkoSonic Endovascular System (EKOS), FlowTriever System, Indigo Aspiration System, and emerging devices like the BASHIR catheter have demonstrated safety and efficacy in clinical trials. Notably, randomized evidence from trials like PEERLESS [81] and upcoming data from HI-PEITHO [16] will help clarify the optimal role of these interventions in guideline-based practice.

APE treatment is complex and should be formulated by an experienced interdisciplinary clinician team and based on careful risk stratification, including different factors, such as patient characteristics, cardio-pulmonary reserve, bleeding risk, and available interventional resources.

Recent guidelines (ESC 2019, ACCP 2021, and the PERT Consortium recommendations) [72,77] continue to support systemic thrombolysis as the first-line reperfusion therapy for high-risk PE, reserving CDT for specific clinical scenarios. Low-dose systemic tPA has emerged as a promising treatment option for acute pulmonary embolism (PE), particularly in intermediate-risk patients. Risk stratification remains the cornerstone of APE management, but as reviewers appropriately noted, definitions for acute right ventricular dysfunction lack standardization, and the prognostic distinction between intermediate–low- and intermediate–high-risk groups remains debated.

## 9. Limitations

As a narrative review inherently carries selection bias, studies were chosen based on relevance and author expertise rather than a systematic or meta-analytic methodology. Although we endeavored to present balanced and current evidence, some emerging technologies and trials may have been inadvertently overlooked.

Moreover, much of the supporting evidence for newer imaging modalities like PC, DDR, and AI-based tools derives from single-center studies or early-phase trials with limited patient cohorts. Consequently, the generalizability of these findings to broader clinical practice remains uncertain.

Finally, interventional radiology techniques, while increasingly utilized, lack extensive randomized controlled trial data in intermediate-risk PE populations, with most published studies being observational or single-arm registries. The forthcoming results from pivotal trials such as HI-PEITHO will be crucial in refining recommendations and clinical indications.

## 10. Conclusions

APE is a complex and potentially life-threatening condition that demands a multidisciplinary and personalized approach. Recent advances in imaging technologies have improved diagnostic accuracy while minimizing patient risk. Minimally invasive endovascular therapies are becoming increasingly central for managing high- and intermediate-risk cases, offering effective alternatives to systemic thrombolysis. Pulmonary Embolism Response Teams facilitate tailored decision-making and improve outcomes through structured collaboration. Continued integration of artificial intelligence, risk stratification models, and clinical expertise will be critical to advancing APE care and improving long-term patient outcomes.

## Figures and Tables

**Table 1:** Advantages and disadvantages of the different imaging techniques in APE diagnosis. CTPA: Computed Tomography Pulmonary Angiography; VMI: Virtual Monochromatic Image; VNC: Virtual Non-Contrast; RV: Right Ventricle; APE: Acute Pulmonary Embolism.

Technique	Pros	Cons
High-Pitch CT	- Very fast scan time (<1 s) - Low radiation dose (~30% lower than standard CTPA) - Lower contrast volume (20 mL) - Reduced motion artifacts - Good for dyspnoic or uncooperative patients - Excellent image quality in >90% cases	- Slightly lower contrast opacification and increased noise in segmental arteries
Dual-Energy CT (DECT)	- Combines anatomical and perfusion imaging - Enables iodine perfusion maps, VMIs, VNC - Detects subsegmental emboli better - Can reduce need for additional imaging - High sensitivity/specificity when combined with advanced post-processing - Allows contrast and dose reduction - Helps assess severity (e.g., RV dysfunction correlation)	- Variable diagnostic performance depending on protocol and post-processing - No substantial advantage over SECT in meta-analysis - Complexity and cost of post-processing - Limited widespread availability and standardization
Photon-Counting CT (PC)	- Superior spatial and contrast resolution - Significantly lower contrast and radiation doses (up to 50% reduction) - Improved image quality and emboli conspicuity - Effective for subsegmental APE detection - Less electronic noise - Compatible with high-pitch and spectral modes - Approaching clinical use in 2025	- Expensive and limited current availability - Few studies report sensitivity/specificity against gold standard - Clinical validation still ongoing despite promising results
Dynamic Digital Radiography (DDR)	- No contrast medium required - Very low radiation dose (~0.2 mSv) - Simple, quick, and non-invasive - Useful in patients with contrast allergies or renal impairment - Effective for functional assessment - Portable and usable bedside	- Limited spatial resolution - Not yet standard for APE diagnosis - Mainly validated for chronic thromboembolic disease - Primarily functional, not anatomical imaging

**Table 2:** This table provides a summary based on the specific performance metrics (Sensitivity, Specificity, Area Under the Curve—AUC) available for each mentioned AI tool.

Name of the Tool	Specificity	Sensitivity	AUC
CINA-PE (Avicenna.AI) [57,58]	94.8%	93.9%	AUC: 0.92
Aidoc [60]	95.8%	92.6%	AUC: 0.87
Retina U-Net/nnDetection (unenhanced CT) [56]	N/A	Varies by location: Central 54.5%, Segmental 81.9%, Subsegmental 80.0%	N/A
Deep Learning Algorithm (Liu et al.) [52]	76.5%	94.6%	AUC: 0.92
Deep Learning Model (Huhtanen et al.) [51]	86.6%	93.5%	AUC: 0.91
End-to-end DL Model (Huang et al.) [48]	N/A	N/A	AUC: 0.84–0.85
Deep Learning Algorithm (Li et al.) [59]	95.5%	92.7%	AUC: 0.95
nnU-Net-based Algorithm (Kahraman et al.) [53]	94.6%	96.1%	N/A

## Data Availability

No new data were created or analyzed in this study. Data sharing is not applicable to this article.

## Abbreviations

The following abbreviations are used in this manuscript:

APE	Acute Pulmonary Embolism
CTPA	Computed Tomography Pulmonary Angiography
DECT	Dual-Energy Computed Tomography
PC	Photon Counting
DDR	Dynamic Digital Radiography
RV	Right Ventricle
VNC	Virtual Non-Contrast
VMIs	Virtual Monoenergetic Images
EID-CT	Energy-Integrating Detector CT
PCDs	Photon-Counting Detectors
DL	Deep Learning
CNNs	Convolutional Neural Networks
CAD	Computer-Assisted Detection
ML	Machine Learning
AUC	Area Under the Curve
DNN	Deep Neural Network
NLP	Natural Language Processing
CT	Computed Tomography
PE	Pulmonary Embolism
V/Q	Ventilation/Perfusion (referring to V/Q scanning)
CTEPH	Chronic Thromboembolic Pulmonary Hypertension
SECT	Single-Energy CT
LB	Linear Blending
RV/LV	Right Ventricle-to-Left Ventricle diameter ratio
